# Comparative Genomics of the Genus Pseudomonas Reveals Host- and Environment-Specific Evolution

**DOI:** 10.1128/spectrum.02370-22

**Published:** 2022-11-10

**Authors:** Zaki Saati-Santamaría, Riccardo Baroncelli, Raúl Rivas, Paula García-Fraile

**Affiliations:** a Departamento de Microbiología y Genética, Universidad de Salamanca, Salamanca, Spain; b Institute for Agribiotechnology Research (CIALE), Villamayor, Salamanca, Spain; c Institute of Microbiology of the Czech Academy of Sciences, Vídeňská, Prague, Czech Republic; d Department of Agricultural and Food Sciences (DISTAL), University of Bologna, Bologna, Italy; e Associated Research Unit of Plant-Microorganism Interaction, USAL-CSIC (IRNASA), Salamanca, Spain; Institut Pasteur

**Keywords:** *Pseudomonas*, environmental microbiology, genomics, host-cell interactions, microbial ecology

## Abstract

Each Earth ecosystem has unique microbial communities. Pseudomonas bacteria have evolved to occupy a plethora of different ecological niches, including living hosts, such as animals and plants. Many genes necessary for the Pseudomonas-niche interaction and their encoded functions remain unknown. Here, we describe a comparative genomic study of 3,274 genomes with 19,056,667 protein-coding sequences from Pseudomonas strains isolated from diverse environments. We detected functional divergence of Pseudomonas that depends on the niche. Each group of strains from a certain environment harbored a distinctive set of metabolic pathways or functions. The horizontal transfer of genes, which mainly proceeded between closely related taxa, was dependent on the isolation source. Finally, we detected thousands of undescribed proteins and functions associated with each Pseudomonas lifestyle. This research represents an effort to reveal the mechanisms underlying the ecology, pathogenicity, and evolution of Pseudomonas, and it will enable clinical, ecological, and biotechnological advances.

**IMPORTANCE** Microbes play important roles in the health of living beings and in the environment. The knowledge of these functions may be useful for the development of new clinical and biotechnological applications and the restoration and preservation of natural ecosystems. However, most mechanisms implicated in the interaction of microbes with the environment remain poorly understood; thus, this field of research is very important. Here, we try to understand the mechanisms that facilitate the differential adaptation of Pseudomonas—a large and ubiquitous bacterial genus—to the environment. We analyzed more than 3,000 Pseudomonas genomes and searched for genetic patterns that can be related with their coevolution with different hosts (animals, plants, or fungi) and environments. Our results revealed that thousands of genes and genetic features are associated with each niche. Our data may be useful to develop new technical and theoretical advances in the fields of ecology, health, and industry.

## INTRODUCTION

Pseudomonas is a large bacterial genus whose members are adapted to live in many diverse biological niches, such as plants (1–3), mammals (4), reptiles (5, 6), insects (7–10), nematodes (11), humans (12), rivers (13, 14), soils (13), and anthropogenic environments (15), among others (16). Due to the ecological, clinical, and biotechnological importance of Pseudomonas bacteria, many research efforts target their functions, such as those involved in the modulation of nutrient cycles (17–19) or the production of secondary metabolites (20, 21) and those responsible for their behavior as beneficial or pathogenic commensals of higher hosts (1, 22–25). Despite this information, many genes and metabolic pathways of Pseudomonas remain undescribed, as does much of the genetic basis of its adaptation and specialization to different lifestyles.

Discovering novel microbial functions is a complex task, for which laborious wet lab experiments are usually required (i.e., *in vivo* transcriptome sequencing [RNA-seq], transposon mutagenesis and phenotype evaluation, and targeted gene editing) (22, 26–29). Even so, these techniques may cause large studies to yield undesired results when the targeted gene/pathway is not properly selected or the first hypothesis is not adequate. Conversely, comparative genomics is rising as a powerful methodology that helps to unveil genes associated with phenotypes or ecological features, reducing research risks and aiding in relevant breakthroughs in understanding microbe mechanisms (30–33). The reduced sequencing costs and the development and easy use of DNA databases have allowed the scientific community to sequence and share thousands of microbial genomes worldwide. Thus, the study of publicly available genomes helps reveal microbial evolution and adaptation in a low-cost and profitable way.

We aimed to explore the potential ecological functions of Pseudomonas and its genomic adaptability to diverse lifestyles and to discover novel genes and functions that participate in the interaction of these bacteria with the environment. We created a database of high-quality publicly available genomes of 3,274 Pseudomonas strains with known isolation sources. Then, we used pangenomic and comparative genomic strategies to find differential features among genomes grouped by habitat or host. This work is reinforced by the large genome data set of closely related strains used, providing powerful findings that advance the knowledge of Pseudomonas ecology and evolution.

## RESULTS

### Obtaining a curated pangenome from high-quality Pseudomonas genomes.

**FIG 1:**
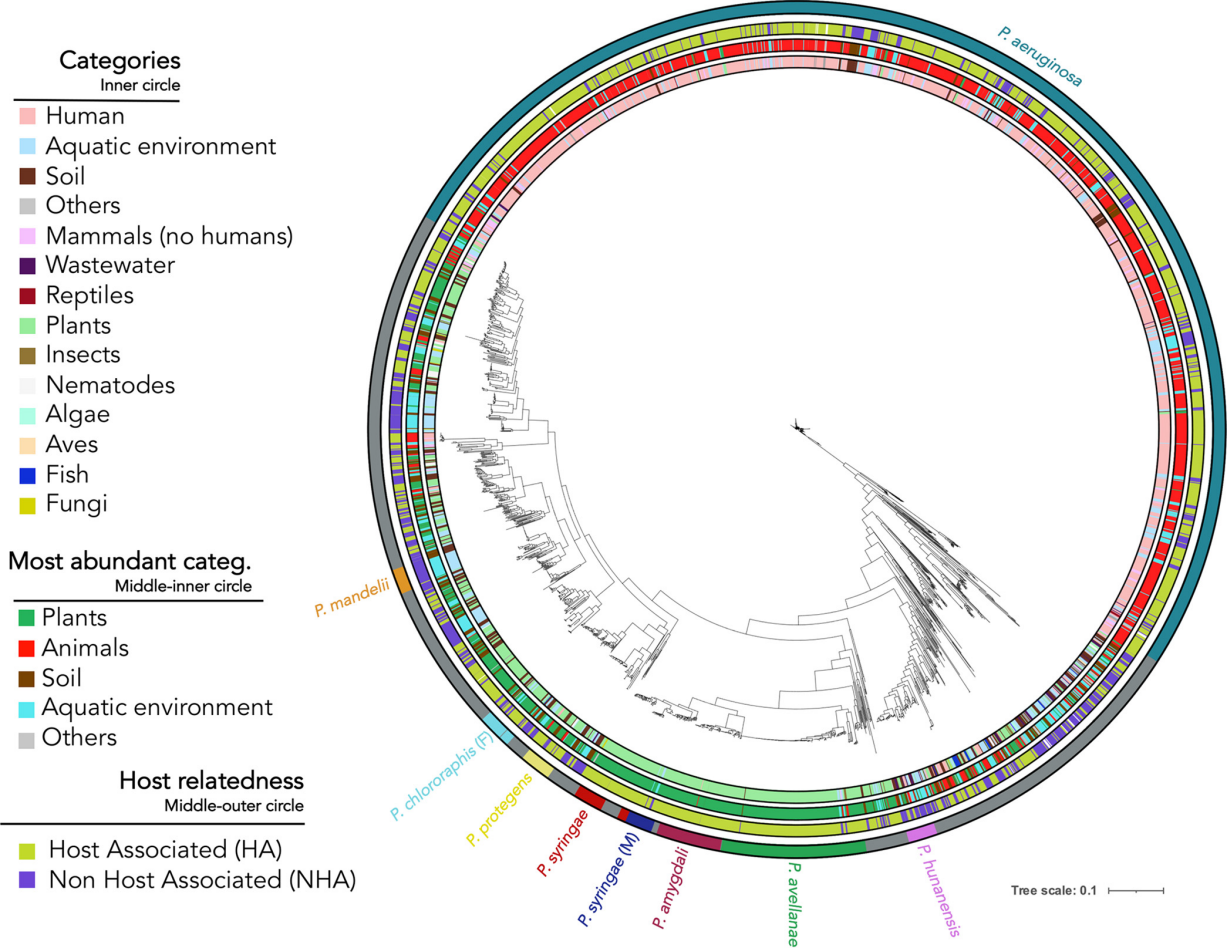
Phylogeny and niche distribution of 3,274 Pseudomonas genomes. The phylogenetic tree was built with the 3,274 Pseudomonas genomes depicting the isolation source for each strain. Each isolation source is labeled with a different color. There are 3 differently colored circles that represent the isolation sources at different levels. The inner circle includes all the different categories. In the following circle, these categories are merged into 5 categories. Then, the third circle comprises the host relatedness (yes versus no). Finally, the outer circle shows the most represented species (>30 genomes) in this genome collection according to the GTDB.

With the aim of studying genes or functions related to the adaptation of Pseudomonas to different hosts or niches, we retrieved 11,167 Pseudomonas genome sequences from public databases. These genome sequences were filtered to retain only high-quality genomes from Pseudomonas strains for which the isolation origin is available publicly. Genomes too phylogenetically distant from the remaining Pseudomonas genomes (there were some clades that were located in an extremely far branch, see the GitHub repository for more details online at https://github.com/zakisaati/Pseudomonas_pangenome) were also eliminated due to their possible misidentification. This filtering led to 3,274 high-quality genomes with trustworthy metadata (Materials and Methods; Fig. 1; see Table S1 in the supplemental material). These genomes have a mean coding region density of 71%, 129 contigs, an *N*_50_ value of 1,042,151 and *L*_50_ value of 15 (Table S1) and were classified in 393 different species according to the Genome Taxonomy Database (GTDB). Pseudomonas aeruginosa is the most represented species (*n* = 1,661), followed by Pseudomonas avellanae (*n* = 177), Pseudomonas amygdali (*n* = 78), and Pseudomonas syringae (*n* = 50). A total of 26 species encompass ≤49 and ≥10 genomes of the data set. A total of 170 species have ≤9 and ≥2 representatives. The remaining 192 genomes belong to unique species. Notably, this analysis is based on the GTDB nomenclature, which splits some valid species into several groups, considering that some strains should represent different taxa. In case those groups were categorized into the current valid taxonomic names, a few species would be more represented. For instance, Pseudomonas fluorescens groups would sum 103 genomes; P. syringae, 93 genomes; Pseudomonas chlororaphis, 73 genomes; Pseudomonas putida, 48 genomes; and Pseudomonas stutzeri, 39 genomes; among others. Of those strains belonging to the same species, only 71 share average nucleotide identity (ANI) values of >99,999% (ANI matrixes are available at Zenodo, https://doi.org/10.5281/zenodo.7105218).

We built a Pseudomonas pangenome, obtaining 326,707 protein clusters (fasta file available at Zenodo, https://doi.org/10.5281/zenodo.7105218) from a total of 19,056,667 coding sequences (CDSs) (70% similarity, 80% coverage). We found a very narrow core genome comprising 65 genes, while most of the genes shaped the accessory genome (see Table S2 in the supplemental material). Each genome has 1,085 to 1,645 soft-core genes (genes present in the 95% of the genomes), which represents 18 to 43% of the total number of genes. The pangenome curve did not reach a plateau, suggesting that a fraction of the diversity of Pseudomonas genes remains cryptic (curve available at Zenodo, https://doi.org/10.5281/zenodo.7105218).

### Divergent niche specialization in Pseudomonas.

We manually classified the genome collection into several categories based on the isolation source (Fig. 1; see Table S3 in the supplemental material). This classification also included broad categories comprising different isolation sources, such as “animals,” “host associated,” (HA), and “non-host associated” (NHA).

Next, we constructed a phylogenomic tree to determine whether the different genomic categories showed evolutionary divergence related to the isolation origin. As shown in Fig. 1, some phylogenetic clades, mainly those including human-related and some plant-related Pseudomonas genomes, were associated mostly with specific environmental niches. This finding suggests that species from these clades experienced specialization events related to their association with hosts, while some other categories may be represented by less niche-specific species. Indeed, the number of genes per genome differed significantly among the isolation sources (see Fig. S1 in the supplemental material).

**FIG 2:**
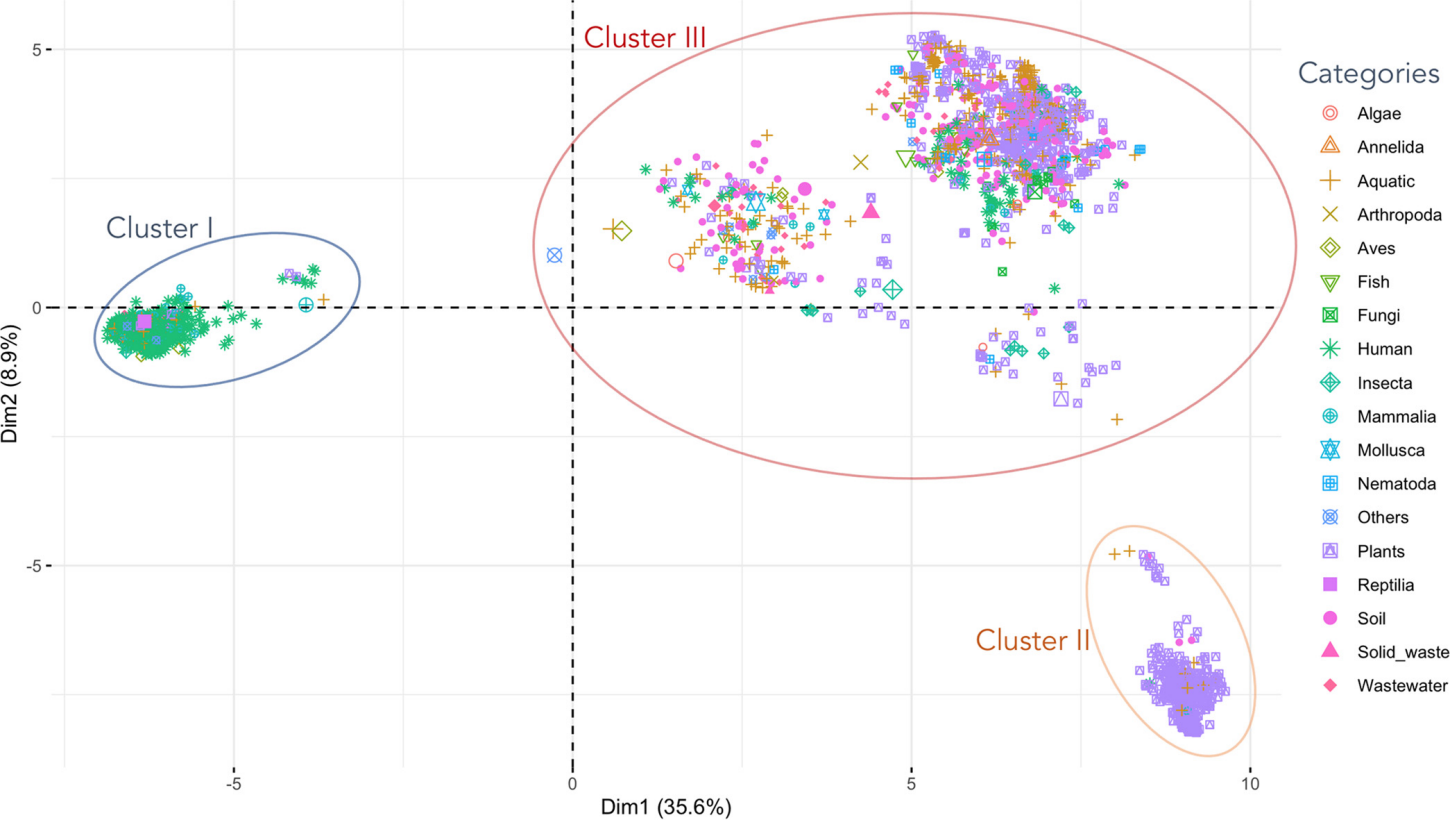
Pseudomonas genomes sharing similar habitats have functional resemblance. The principal-component analysis (PCA) is based on the nonredundant presence of Clusters of Orthologous Groups of proteins (COGs) of each of the 3,274 Pseudomonas genomes of this study. Each symbol represents an isolation category. We depict 3 main functional clusters of genomes that comprise (i) human-related Pseudomonas, (ii) some plant-related Pseudomonas (mainly epiphytic and phytopathogenic strains), and (iii) the remaining Pseudomonas genomes.

This evolutionary tendency was also preserved in regard to the functional (Clusters of Orthologous Genes [COG] content) profiles of Pseudomonas (Fig. 2; see Fig. S2 in the supplemental material). We found 3 main functional clusters of genomes that comprised (i) human-related Pseudomonas, (ii) some plant-related Pseudomonas, and (iii) the remaining Pseudomonas genomes. Interestingly, the second genome cluster included epiphytic and phytopathogenic Pseudomonas, while rhizospheric and plant-beneficial Pseudomonas members were included in the third and most diverse cluster (Fig. 2).

### Diversity of Pseudomonas functions.

**FIG 3:**
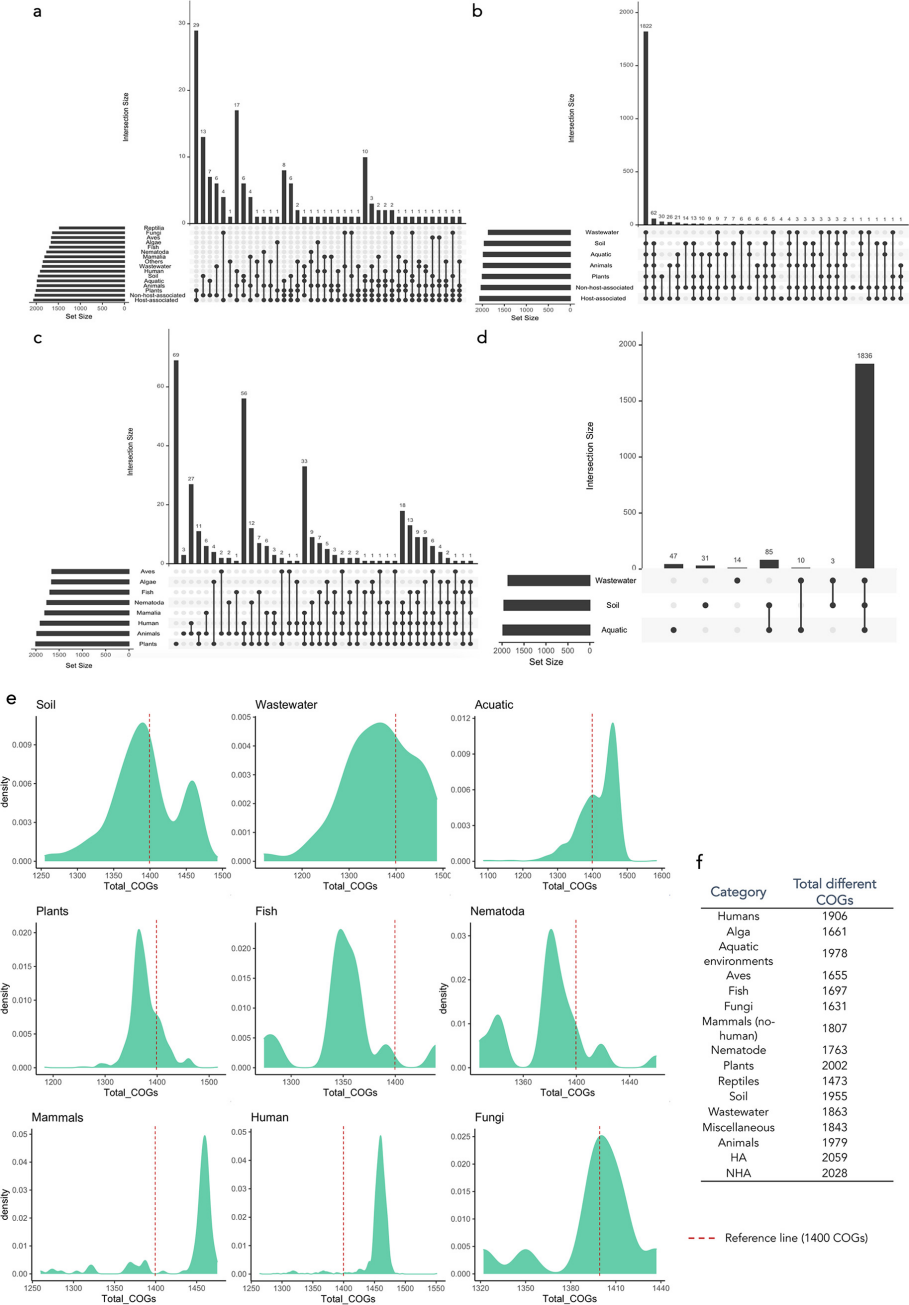
The lifestyle of Pseudomonas strains is a key factor that determines their metabolic range. (a, b, c, d) Intersection plots showing the number of nonredundant Clusters of Orthologous Groups of proteins (COGs) in each category and the unique COGs shared by two or more categories. (e) Density plots relating the number of genomes with the COG content. The red dashed line represents 1,400 COGs as a reference to compare different plots. (f) Table of total unique COGs within each isolation source category.

We examined the diversity of nonredundant COG functions within each of the genome categories. HA genomes are slightly more functionally diverse (higher number of nonredundant COGs) than NHA genomes (Tukey honestly significant difference [HSD], adjusted *P* value [p-adj] = 2.22e-16) (Fig. 3; see Table S4 in the supplemental material). The genomes with the highest COG diversity are those within the categories “human” and “mammals.” Plant-associated Pseudomonas strains are less functionally diverse than those associated with mammals, humans, fungi, and soil (Tukey HSD, p-adj < 0.05); however, this category encompasses the highest diversity of unique functions (2,002 different COGs) (Fig. 3).

Despite the considerable amount of genetic diversity found in the pangenome analyses of Pseudomonas, where we found a small core pangenome (65 genes present in all the genomes), 1,822 COGs were shared among the broadest categories (animals, plants, HA, NHA, aquatic environments, soil, and wastewater) (Fig. 3), which implies a large core functional pangenome (COG functions present in all the strains) in Pseudomonas. This result means that there is a large genetic variability in those Pseudomonas genes that are related with core metabolic functions.

### Insights into the metabolism of carbohydrates and proteins.

**FIG 4:**
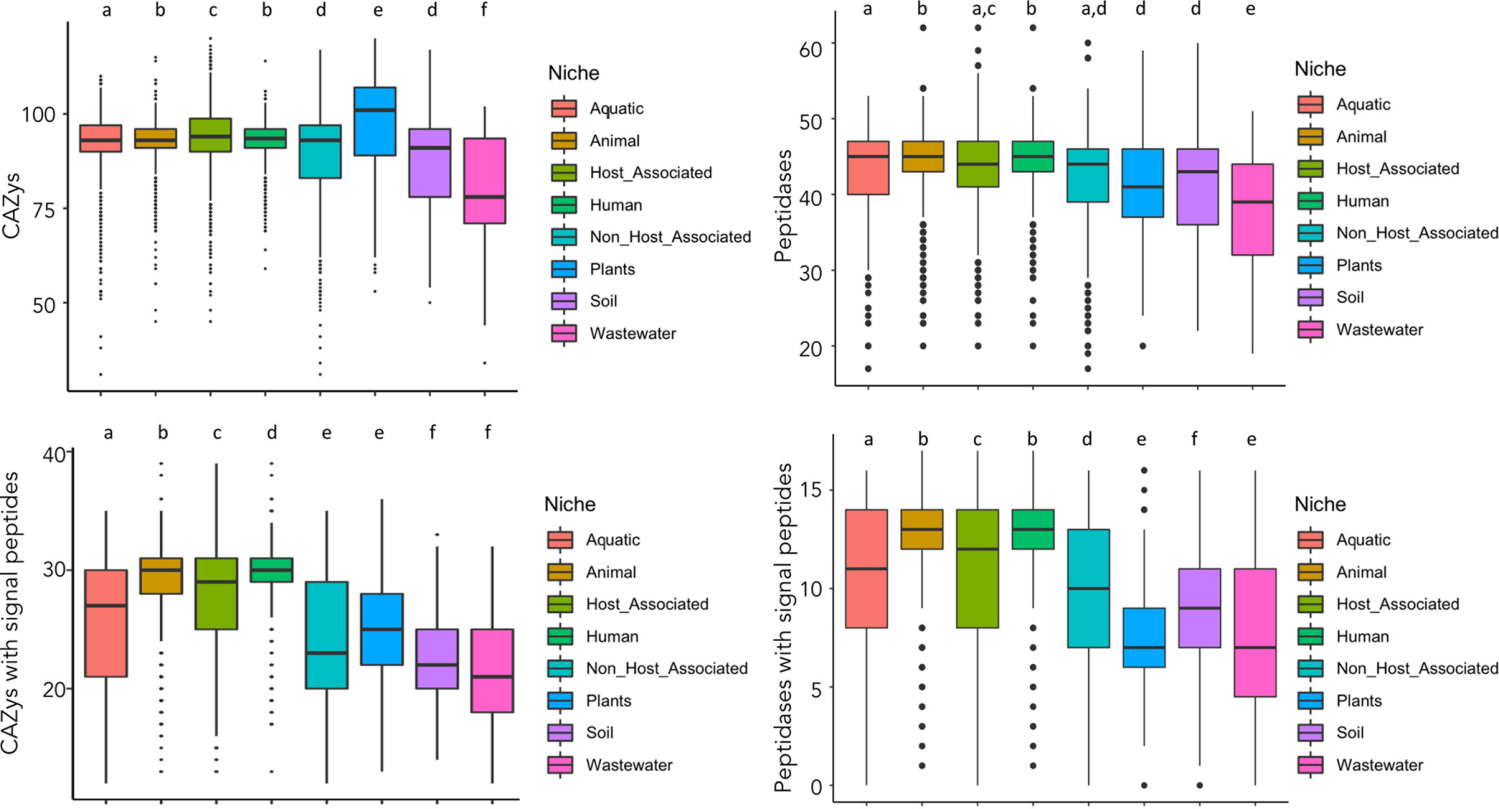
The potential to metabolize carbohydrates and proteins is shaped by the isolation source. This figure includes boxplots that represent the content of either CAZy and peptidase per each genome and isolation source, as well as the enzymes with signal peptides. Different letters represent groups with significant differences (p adj < 0.05).

Carbohydrates are the main carbon source for most living organisms. The ability to use carbohydrates present in a certain niche is crucial for the successful adaptation of most bacteria to a given ecosystem. Each host or environment harbors different amounts and diversities of carbohydrates. We aimed to study the potential of the different Pseudomonas strains to metabolize carbohydrates. To do so, we annotated the genomes with the Carbohydrate Active EnZymes (CAZy) database, which returned a total of 305,915 proteins involved in carbohydrate metabolism (see Table S5 in the supplemental material). Our results suggest a niche-dependent potential to metabolize these compounds (Fig. 4). The Pseudomonas strains isolated from plants, which are hosts with large amounts of complex carbohydrates, showed a higher CAZy content (>100 CAZys/genome), while wastewater-associated Pseudomonas represented the category with the lowest number of CAZys (<80 CAZys/genome) (Fig. 4).

Peptidases catalyze the cleavage of a vast variety of peptides and are associated with many diverse biological functions. Thus, the adaptability of bacteria can be influenced by this proteolytic ability. Here, we show that the lifestyle of the Pseudomonas strains significantly influences the peptidase content (Fig. 4). This finding denotes that the mean content of peptidases on the studied strains is significantly related to the isolation source (Tukey HSD; p-adj < 0.05). For example, the strains living in wastewater environments carry a lower number of peptidases than those isolated from other niches (Fig. 4).

Both CAZys and peptidases can be secreted or placed within the outer membrane, enabling a broader set of interactions with the environment and surrounding microbes. Signal peptides are markers that allow proteins to enter secretion pathways. Consequently, the presence of signal peptides on these enzymes can be crucial for the adaptability, fitness, and interaction of Pseudomonas within its ecosystem. Interestingly, despite having the highest CAZy content, plant-associated Pseudomonas showed far fewer secreted CAZys than the strains belonging to the animal, human, and aquatic categories (Fig. 4). Similarly, the presence of peptidases with signal peptides per genome was the lowest in the group “plants” (Fig. 4). This result suggests that plant-associated Pseudomonas strains use most of their degradative arsenal to metabolize carbohydrates and peptides in the cytoplasm in order to obtain energy, carbon, and nitrogen, while a larger proportion of enzymes with signal peptides encoded by the other strains may be dedicated to interactions with the environment.

### Stress resistance.

In certain environments, bacterial survival requires resistance to environmental threats (heat, presence of antibiotics, metals, and biocides; see Materials and Methods). Thus, we looked for mechanisms enabling Pseudomonas to resist these stress conditions, and we analyzed their distribution (see Table S6 in the supplemental material). Among the Pseudomonas strains studied in this work, those isolated from humans possess the most genetic machinery related to the mentioned threats. In contrast, plant-associated strains possessed the smallest number of stress resistance mechanisms, even smaller than the number observed for strains isolated from bulk soil (p adj < 0.05) (see Fig. S3 in the supplemental material), suggesting that the plant environment protects bacteria from environmental stresses or microbiological competition. Overall, HA Pseudomonas have a broader resistance potential (mainly antimicrobial resistance) than NHA strains (p adj < 0.05).

### Thousands of proteins and functions show specific associations with the environment in Pseudomonas.

Each microenvironment where Pseudomonas bacteria live has unique physicochemical characteristics, available molecules for bacterial nutrition, or, in the case of host-related niches, defense mechanisms. To adapt to the different natural conditions of the niches they inhabit, Pseudomonas bacteria should have undergone adaptation events driven by the evolution of their accessory genome, offering some survival advantage. We used a comparative genomics approach to study protein and functional (COGs, resistance-related proteins, and CAZys) enrichment in each niche/isolation category. We used Scoary to detect specific enrichments, yielding thousands of proteins and functions associated with different niches or hosts (Table 1; see Files S3 to S6 in the supplemental material).

**TABLE 1:** Number of proteins or functions associated with the isolation origin of Pseudomonas strains

Category	No. of:			
	Protein clusters^*a*^ (HP^*b*^)	COGs^*a*^	Resistance-related proteins^*c*^	CAZys^*c*^
Aquatic environment	4.841 (3.327)	93	16	145
Fish	663 (472)	3	1	34
Fungi	263 (115)	7	4	53
Humans	7.060 (4.308)	465	58	108
Mammals (nonhuman)	2.563 (1.337)	81	6	64
Nematodes	677 (409)	24	2	54
Plants	10.916 (7.214)	211	12	344
Soil	3.476 (1.767)	142	13	254
Insects	77 (19)	-	2	96
Wastewater	524 (283)	17	7	70
Animals	6.837 (4.202)	431	55	107
Host associated	6.975 (4.173)	315	34	162
Not host associated	9.099 (5.382)	225	20	309

a *P* < 10^−6^ (Benjamini-Hochberg correction).

b HP, hypothetical protein.

c *P* < 10^−2^ (Benjamini-Hochberg correction).

We found that a large proportion of the proteins detected with Scoary that seem to be related to the isolation source do not have any functional annotation (hypothetical proteins). Additionally, considering host relatedness, we found that more proteins were associated with the NHA category, while the number/diversity of COGs and resistance-related proteins associated with hosts (HA) was higher. Focusing on clear isolation sources, the genomes isolated from plants were associated with the most proteins. Similarly, human-related strains showed more unique associations of COGs and stress resistance-related proteins. Additionally, Pseudomonas strains isolated from plants, soils, and natural aquatic environments were associated with the highest number of CAZys (Table 1; Table S5). Many protein clusters are significantly associated with more than one niche (see Fig. S4 in the supplemental material). For instance, 4,052 proteins are associated with “animals,” “humans,” “mammalia,” and “hosts,” probably due to a bias toward the higher number of human isolates present in our animal/mammalia/host data sets which implies an overlap between these niches. Similarly, 165 proteins are significantly associated with both “soil” and “plants” categories, which could be due to isolation or metadata bias, or even due to some role in the rhizosphere environment. We also compared the proteins associated with HA and NHA categories and found substantial similarity (see Fig. S5 in the supplemental material), suggesting that these associations do not include large groups of extremely evolutionarily distant proteins.

**FIG 5:**
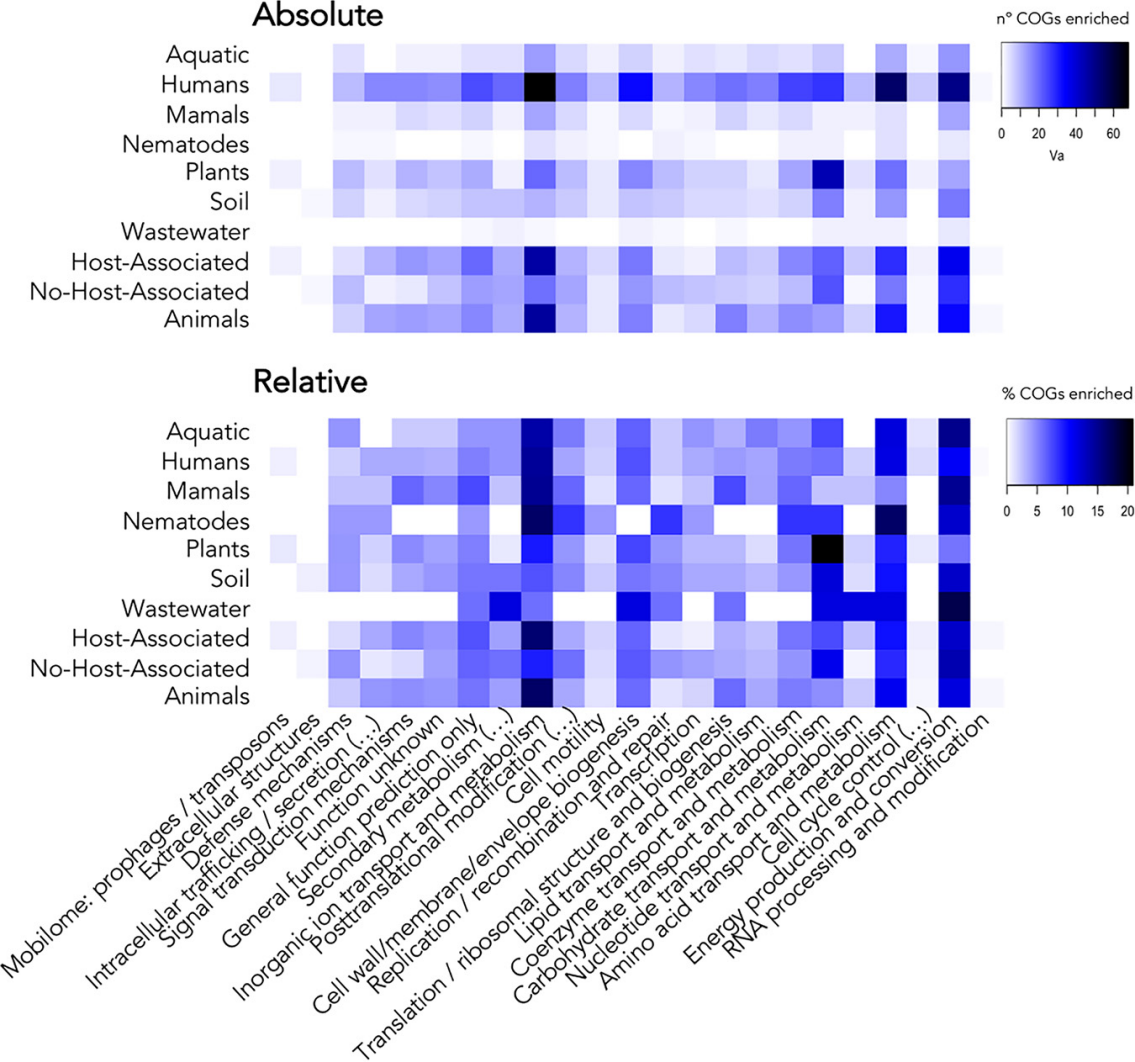
The metabolic profile based on the niche-associated COGs in Pseudomonas reveals metabolic categories enriched in each group of genomes. The heatmaps represent the different categories of Clusters of Orthologous Groups of proteins (COGs) enriched in Pseudomonas genomes. At the top is shown the sum of significantly enriched COGs falling within a certain category. At the bottom, each count of COGs has been relativized to the total number of COGs associated with each host or niche.

Focusing on the metabolic pathways or metabolism categories in which niche-associated COGs are classified (Fig. 5), we show that carbohydrate metabolism is specifically enriched in plant-associated strains. Additionally, human- and aquatic environment-related Pseudomonas strains have a large proportion of enriched COGs associated with the transport and metabolism of inorganic ions. The largest proportion of HA COGs was observed for energy metabolism or the metabolism of inorganic ions and amino acids.

Additionally, among the enriched proteins mentioned above, some have already been suggested to function within Pseudomonas habitats. For example, the HCN-ABC operon appears to be associated with humans (34), the metapyrocatechase enzyme (also named catechol 2,3-dioxygenase, responsible for toluene degradation) (35) was found to be enriched in wastewater environments, and 1-aminocyclopropane-1-carboxylic acid (ACC) deaminase is enriched in Pseudomonas strains isolated from plant environments (36) (File S3). Additionally, we found proteins involved in vitamin B12 synthesis associated with insect niches; B12 supply has been suggested to play an endosymbiont role in insects (37, 38), but this role has never been proven in Pseudomonas.

In contrast, many of the genes found to be enriched in some groups of Pseudomonas have never been described as relevant for interactions with the environment or the host. For instance, we found that a protein similar to the biofilm dispersion protein (BdlA) is strongly enriched in those isolates associated with nematodes (Benjamini-Hochberg’s [B-H] p-adj = 4.39 × 10^−10^), which suggests its potential importance for the removal of biofilm in these hosts. Similarly, the YdeP protein, which is suggested to be related with acid resistance, is enriched in the isolates associated with insects (B-H p-adj = 7.86 × 10^−14^). The protein with the best significant association to plant-niche encodes an L-glyceraldehyde 3-phosphate reductase (B-H p-adj = 2.11 × 10^−305^). Similarly, the enrichment of a blue-light photoreceptor in soil associated pseudomonads (B-H p-adj = 8.57 × 10^−51^) might be linked with the dynamics or the response of bacterial cells to the soil depth. More examples are the YjcH protein and a xanthine permease in human-associated Pseudomonas, the transcriptional regulator *XynR* in plant-associated strains, and an operon homologous to the Yop virulon in fish-related strains, or in a broader sense, any of the thousands of hypothetical proteins associated with each niche.

### Genetic dynamics.

**FIG 6:**
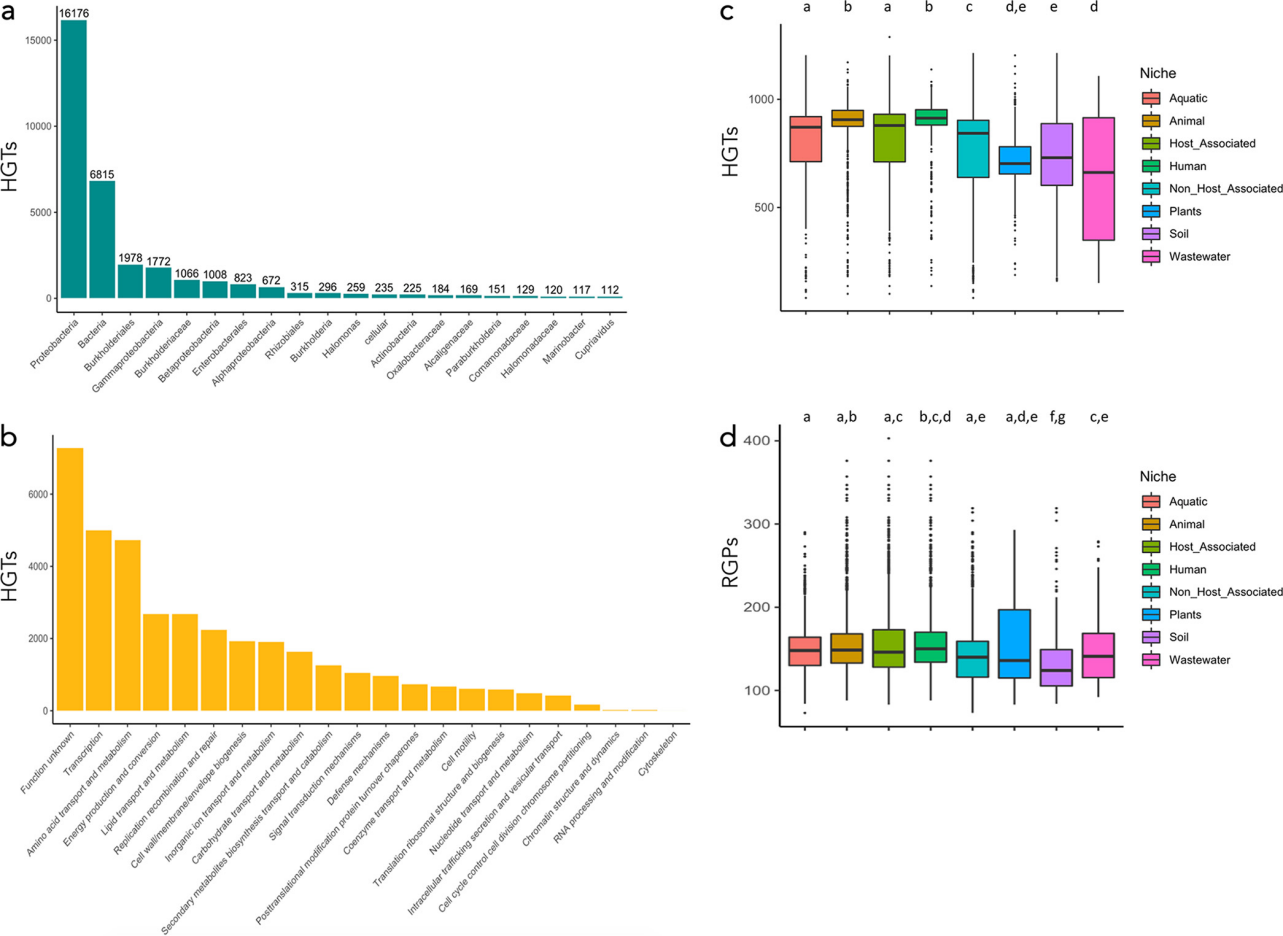
Gene dynamics in the Pseudomonas genomes. (a) This figure represents the number of detected HGT genes classified based on the taxa that is supposed to be the source of the horizontal transfer. Only the 20 most abundant taxa are displayed. (b) Bars represent the number of genes derived from HGT events according to the categories in which the encoded protein is classified based on the COG annotation. (c, d) Boxplots showing the number of HGT and RGP events per genome and classified into the different isolation sources. Different letters represent groups with significant differences (p adj < 0.05).

Since horizontal gene transfer (HGT) influences bacterial adaptability to novel environmental conditions, we searched for Pseudomonas genes that may have been acquired from other microbial taxa via this process. A total of 11.5% of the representative sequences of the Pseudomonas pangenome were detected as potential horizontally transferred genes. Of these genes, the majority could have a bacterial origin, primarily from *Proteobacteria*, followed by *Burkholderiales* (Fig. 6). Additionally, there are also proteins for which the origin is suggested to be domain *Archaea*, superphylum *Terrabacteria*, or phylum *Firmicutes*, among other groups, even though these proteins are present in a smaller proportion within the pangenome (Fig. 6).

The composition of the different microbiomes or the features of a certain niche may imply differential dynamics in the transfer of genes. In this regard, HA strains of Pseudomonas showed slightly higher gene transfer rates than NHA strains (Fig. 6), which may be influenced by the antibiotic resistance spread of P. aeruginosa (HA) strains. The most notable difference is that between the animal (mean, 885 HGTs/genome) and plant (mean, 718 HGTs/genome) categories (Tukey HSD p-adj = 1.62 × 10^−11^). The Pseudomonas strains isolated from soils and wastewater showed high variability in their HGT content (Fig. 6).

With regard to the functional annotation of the representative genes/proteins of the pangenome likely acquired by HGT events, 134 were annotated as resistance-related proteins (55.4% of the total number of resistance-related proteins detected). Moreover, several invertases (proteins that switch antibiotic-resistance regulatory genes, among other functions [39]) appear to be acquired from Klebsiella species. Similarly, 850 of 3,245 CAZys would have been horizontally acquired. Additionally, the classification of these proteins within COG categories reveals that most of them do not have an assigned functional category, whereas the assigned proteins belong mainly to the “transcription,” “amino acid and transport metabolism,” “energy production and conversion,” and “lipid transport and metabolism” categories (Fig. 6).

Commonly, clusters of genes acquired by HGT are located in regions of genome plasticity (RGPs), such as genomic islands or plasmids. We looked for RGPs through the graphical and partition-driven methods implemented in PPanGGOLiN (Materials and Methods). We detected 499,509 RPGs in total (152,6 RGPs/genome). We found that lifestyle impacts the RGP content per genome (Fig. 6). The Pseudomonas strains isolated from soils showed the fewest RGPs, those isolated from humans had intermediate numbers of RGPs/genome, and those isolated from plants had the most RGPs.

## DISCUSSION

Earth ecosystems and living organisms (hosts such as animals or plants) are strongly influenced by their microbiomes (40–43). Thus, understanding the functions of bacteria in their biological niches is of utmost importance and can be profitable for the study of animal or plant illnesses and for the development of biofertilizers and bioremediation agents, among others. (3, 9, 27, 44, 45). A common integrant of the microbiomes inhabiting many diverse habitats and hosts is the genus Pseudomonas (16). The variability of niches where Pseudomonas can survive makes the study of both the ecological functions and the metabolic potential of these bacteria very appealing. To perform deep analyses of the ecological functions of bacteria belonging to this genus, we implemented a comparative genomics study of more than 3,000 Pseudomonas strains, revealing their probable roles in their isolation niches and providing an understanding of their likely evolution and adaptation mechanisms.

We found that plant-associated Pseudomonas strains have smaller genomes than other strains of this genus. This finding may indicate a more intimate cross-kingdom interaction and specialization within these hosts (46). Nonetheless, this tendency does not stand out when HA and NHA genomes are compared. We also found that HGT events affect 10 to 20% of the genes in each Pseudomonas genome. Most of these events involved bacteria from the same phylum (*Proteobacteria*), which usually represents a relevant proportion of the bacterial communities of the common habitats of Pseudomonas (47–52). More than one-half of the detected clusters of resistance genes could have been horizontally acquired. This finding supports those of Freschi et al. (53), who showed that HGT events drive the gain of antibiotic resistance and virulence in P. aeruginosa.

Our findings indicate that strains associated with humans have a larger number of resistance-related genes than other strains. Nevertheless, this difference could be the result of biased annotation; while many P. aeruginosa resistance genes are included in the available databases, the resistance genes belonging to less-studied environmental Pseudomonas strains may have never been elucidated.

Here, we show that plant-related Pseudomonas strains have enriched genetic machinery for carbohydrate metabolism, probably to adapt to the complex carbohydrate content in the plant environment (54, 55). Levy et al. (56) reached similar conclusions when comparing genomes belonging to different phyla associated or not associated with plants. The enrichment of signal peptides among CAZys encoded by animal-related Pseudomonas strains may be due to the involvement of these enzymes in biofilm formation and/or the degradation of host tissues. In contrast, the CAZys of strains isolated from plant hosts seem to be located mainly in the cytoplasm, suggesting an enhanced ability to metabolize carbohydrates for use as a C or energy source. Similarly, we found variation in the content of secreted peptidases in the categories of genomes examined in this work, suggesting a role of these proteins in the lifestyle adaptations of Pseudomonas. These findings agree with those of Nguyen et al. (57), who found segregation of extracellular peptidases of bacteria and archaea according to their habitats.

We found an enrichment of inorganic ion metabolism in human-associated Pseudomonas, which was due mainly to the enrichment of proteins related to H^+^ transport and iron metabolism. The first process may aid in the adaptability of P. aeruginosa to the variability of H^+^ levels in lung tissues (58, 59). Additionally, the enrichment of iron-related functions is related to siderophore production by this pathogenic species, which also serves as a mechanism of niche competition (60).

Adding to previous comparative genomic works aiming to find undescribed microbial genes with ecological functions (31–33, 61, 62), we discovered thousands of such genes that may have key functions in the environmental interaction or the adaptation of Pseudomonas (File S3 to S6). Interestingly, the annotation processes identified many of them as hypothetical proteins. Here, we provide a catalogue of genes with likely relevant roles in the lifestyle of Pseudomonas bacteria, allowing researchers to direct efforts toward deeply investigating their functions and to discover new bacterial functionalities that currently remain hidden (27, 63–65). Those proteins with the highest odds ratio and lowest Benjamini-Hochberg’s (B-H) adjusted *P* value found on each of the tables presented in File S3 should be considered strongly related with the Pseudomonas lifestyle in each niche. Hence, to further use our data, the main findings might be prospected carefully based on the interest of the research. For instance, we suggest using the representative sequences for the Pseudomonas*-*pangenome proteins (https://doi.org/10.5281/zenodo.7105218) to compare (i.e., blastp searches) our data with particular proteins of interest and thereby translate the ecological importance found for the query protein (if any).

Despite the large genome data set, which provides us high statistical confidences, there are some issues that may obscure the results, which are as follows: (i) it is possible that the genome metadata obtained from databases were not sufficiently detailed or even incorrect; (ii) the isolation of bacterial strains in a niche does not always imply a strict adaptation of that strain into the niche, since it may be a transient cell in the environment or even a contaminant; and (iii) biases inherent to *in silico* methods (i.e., difficulty to cluster proteins/genes with similar functions properly) may draw some wrong conclusions. Also, some of the results may be just the consequence of phylogenetic signals that may bias the presence/absence of genes/proteins in some groups of genomes (i.e., the category of human isolates is comprised mainly by P. aeruginosa). Thus, for better confidence to use our results, they should be validated experimentally (e.g., genetic modifications and transcriptional information).

In summary, we show the genomic adaptability patterns of Pseudomonas strains to different lifestyles. For example, plant-associated Pseudomonas strains dedicate the largest number of genes to the metabolism of carbohydrates, but the involved proteins are likely located in the cytosol, in contrast to other strains that present a higher proportion of CAZys in the outer envelope or excrete them. Additionally, contrary to plants, the human/animal environment seems to add pressure to resist stresses, although this issue may be biased toward the better understanding of clinically relevant genes. Furthermore, the association of Pseudomonas with higher hosts increases the probability of gene exchange through horizontal transfer. Overall, our results will facilitate studies focusing on the evolutionary dynamics, ecology, biotechnology, and clinical relevance of bacteria. New insights into genes or functions associated with isolation niches can inspire scientific applications in infectious disease diagnosis and treatment or even the development of engineered strains with biotechnological uses.

## MATERIALS AND METHODS

### Creation of a collection of genomes with a known ecological niche of isolation.

We downloaded a total of 11,167 genomes of the bacterial genus Pseudomonas with their metadata from the JGI-IMG database (https://img.jgi.doe.gov/), Pseudomonas Genome DB (https://www.pseudomonas.com), EzBioCloud Genome Database (https://help.ezbiocloud.net/ezbiocloud-genome-database/), and NCBI genome database (https://www.ncbi.nlm.nih.gov/genome/). We manually inspected the list of genomes to remove all those that did not have concise information about the isolation source or those that, according to the name of the strain, were redundant between genomes from different databases. The quality of the genomes was evaluated with QUAST (v5.2.0) (66) and BUSCO (v5.4.3) (67). Genomes with less than 95% completeness and/or that were highly fragmented were removed for this study. We built a phylogenetic tree with all remaining genomes with the UBCG program (68), which extracts, concatenates, and aligns 92 housekeeping genes from each genome and then builds the tree. UBCG was set to use codon-based alignment with FastTree (maximum likelihood; GTR + CAT model) and a gene support index threshold of 95%. The cutoff for gap-containing positions was set at 50%. The phylogeny was visualized with the iTOL program (69), where genomes that were placed out of the phylogenetic tree (see picture in extended methods described online at https://github.com/zakisaati/Pseudomonas_pangenome) were identified and removed from the analysis. Finally, we created a new phylogenetic tree with the remaining 3,274 genomes, and we depicted the isolation metadata in iTOL.

### Pangenome analysis.

We annotated the 3,274 genomes with Prokka (v1.14.6) (70). Once the genomes were annotated, the files in gene feature format (GFF) were used to perform the pangenome calculations and comparative genomics analyses. To do these analyses, we ran PPanGGOLiN (71) (v1.1.96) following the instructions of the developers and using the default values at each step of the process except for the MMseqs2 (72) clustering of proteins, for which an identity percentage of 70% was chosen. PPanGGOLiN uses a sophisticated method that defines partition nodes to build pangenome graphs used to classify gene families into persistent (conserved in the majority of genomes), shell (present at intermediate frequencies in the genome collection), and cloud (present at low frequency) (see the methods described by Gautreau et al. [71]). Scripts from this program were used to generate a matrix of the presence/absence of protein families. We obtained pangenome statistics from this program. Then, we used the roary_plots.py script (https://sanger-pathogens.github.io/Roary/) to generate graphs of the pangenomes, using the phylogenetic tree built with the UBCG program as the basis.

### Search for protein functions.

We annotated the proteomes with the dbCAN2 program (73) (standalone v2.0.11; https://github.com/linnabrown/run_dbcan) to search for enzymes related to carbohydrate metabolism (CAZys). We retained only the CAZys detected by at least 2 of the 3 algorithms (HMMER, DIAMOND, and HotPep) used by the program. Peptidases were detected through a DIAMOND (74) search (E value threshold, 10^−3^) against the MEROPS database (75). The search for proteins related to resistance to antimicrobials, biocides, and other abiotic stresses was carried out by annotating the representative sequences of the groups of orthologous proteins (obtained with PPanGGOLiN) with the AMRFinderPlus (76) program (v3.9.8). The signal peptide search was performed via SignalP (v5.0b) (77). COG terms were retrieved from Prokka annotations.

### Gene dynamics.

Possible HGT events were estimated using the HGTector program (v2.9b3) (78), with the following flags: method = diamond, E value = 1e-10, and tax-unirank = species. The protein database was compiled from all protein sequences of NCBI RefSeq genomes of bacteria, archaea, viruses, fungi, and protozoa (1 genome per species) plus all NCBI-defined reference, representative, and type material genomes.

We looked for RGPs with the panRGP (79) algorithm implemented within the PPanGGOLiN program (71). These regions correspond to genomic islands, plasmids, and regions that are missing in multiple strains.

### Statistical analyses and measurement of protein enrichment among different isolation categories.

We used the Scoary program (v1.6.16) (80) to study the association of genes, proteins, or functions with the isolation source. To do so, a presence/absence matrix of genes/proteins/functions in each genome and a comma-delimited table (.csv) encoded in binary code (0 and 1) were used as input so that each genome was assigned with corresponding metadata. Statistical calculations were performed using a *P* value adjusted with Benjamini-Hochberg’s (B-H) method for the correction of multiple comparisons (81). The results tables were investigated to select only those data with *P* values (BH correction) lower than the chosen threshold, which was set to 10^−2^ for comparisons of CAZYs and resistance-related genes and 10^−6^ for the protein and COG function analyses. Genes with functions with odds ratios higher than 1 and *P* values less than the selected threshold were considered to be associated with the isolation source.

We searched for significant variation in the number of different functions of the genomes of Pseudomonas associated with distinct niches by using Tukey’s test for multiple comparisons (Tukey HSD) in the analysis of variance (ANOVA) framework with the “*stats*” R package (v4.0.2). The differences were visualized with the ggplot2 (v3.3.2) (82) package for R and the UpSetR package (v1.4.0) (83).

We built a sequence similarity network (SSN) comprising proteins significantly associated with HA or NHA categories with the Enzyme Function Initiative-Enzyme Similarity Tool (EFI-EST) (84). Then, we visualized this SSN in Cytoscape (v3.7.2) (85).

### Taxonomic analysis of the genome collection.

We used the GTDB-Tk program (v2.1.1) (86) to classify each genome into a Pseudomonas species though the “classify_wf” command. This workflow uses the closest ANI value to locate the user strain into the closest species in the GTDB.

We also compared the pairwise similarity of the genomes (all versus all) by measuring the ANI distances with FastANI (v1.33) (87) and adding the “–matrix” flag.

### Data availability.

There are files hosted at Zenodo (https://doi.org/10.5281/zenodo.7105218). This repository includes the following: (i) the pangenome rarefaction curve in html format, (ii) the representative sequences of the protein clusters of the Pseudomonas pangenome, (iii) a folder with the 3,274 genomes of our study, and (iv) two large files which are the output from executing FastANI on the genome collection.

We also included bioinformatic codes and source data in the GitHub repository created for this article (https://github.com/zakisaati/Pseudomonas_pangenome).
