# Supplementary material for: Comparative Genomics of the Genus Pseudomonas Reveals Host- and Environment-Specific Evolution
Source: Microbiol Spectr. 2022 Nov 10;10(6):e02370-22. doi: 10.1128/spectrum.02370-22 (PMC9769992; doi:10.1128/spectrum.02370-22)

Supplementary information for

Comparative genomics of the genus *Pseudomonas* reveals host- and environment-specific evolution

Zaki Saati Santamaría[1,2,3*], Riccardo Baroncelli[4], Raúl Rivas[1,2,5], Paula García-Fraile[1,2,5]

[1]Departamento de Microbiología y Genética, Universidad de Salamanca, 37007 Salamanca, Spain.
[2]Institute for Agribiotechnology Research (CIALE), Villamayor, 37185 Salamanca, Spain.
[3]Institute of Microbiology of the Czech Academy of Sciences, Vídeňská 1083, 142 20 Prague, Czech Republic.
[4]Department of Agricultural and Food Sciences (DISTAL), University of Bologna, 40126 Bologna, Italy.
[5]Associated Research Unit of Plant-Microorganism Interaction, USAL-CSIC (IRNASA), 37008 Salamanca, Spain.
*Corresponding author: zakisaati@usal.es

External data:

There are files hosted at Zenodo (https://doi.org/10.5281/zenodo.7105218). This repository includes: (I) the pangenome rarefaction curve in html format; (II) the representative sequences of the protein clusters of the Pseudomonas pangenome; (III) A folder with the 3,274 genomes of our study and (IV) two large files which are the output from executing FastANI on the genome collection.

We also included bioinformatic codes and source data in the GitHub repository created for this article (https://github.com/zakisaati/Pseudomonas_pangenome).

Content of this PDF:

Captions for supplementary information: page 2
Figure S1: page 3
Figure S2: page 4
Figure S3: page 5
Figure S4: page 6
Figure S5: page 7

Captions

Supplemental Figures

Figure S1. Boxplots of the gene content per genome and isolation source. Different letters represent groups with significant differences (p adj < 0.05).

Figure S2. Principal component analysis (PCA) based on the COG-term content of each of the 3,274 *Pseudomonas* genomes of this study. Colors/symbols represents the isolation source: the red circles indicate strains isolated from hosts (living beings) and the blue triangles, strains not isolated from hosts.

Figure S3. Boxplots of the content (per genome and isolation source) of encoded proteins related to some types of environmental stress resistance. Different letters represent groups with significant differences (p adj < 0.05). The table summarizes the number of each specific type of resistance in the *Pseudomonas* pangenome.

Figure S4. Intersection plot showing the number of significantly enriched proteins in each isolation category and those enriched in two or more categories (connected nodes).

Figure S5. Sequence similarity network (SSN) of the proteins significantly associated (green nodes) and not associated (red nodes) with hosts. Each node represents a cluster of proteins of the *Pseudomonas* pangenome. Those clusters of proteins that are connected with edges share high similarity. Thus, each group of connected nodes represents closely related clusters of proteins. In this case, the main groups encompass both HA and NHA proteins, so there are not big clusters of closely related HA or NHA proteins.

Supplemental Files

Supplemental File 1. Datasheet that includes the following 6 supplementary tables:

Table S1. Metadata, source and features of the 3,274 genomes of our collection.

This table includes the name for each downloaded genome, its isolation source, the database containing this genome and its reference ID, and genomic features (n° contigs, CDSs, genome length and G+C% content).

Table S2. Summary and characteristics of the *Pseudomonas* pangenome.

Number of proteins included in each pangenome PPanGGOLiN partition.

Table S3. Traits (isolation source) of the *Pseudomonas* genomes.

Each isolation category represents a column in which "1" indicates that the genome is characterised by that trait and "0" indicates that it is not.

Table S4. Number of different COGs encoded by each of the 3,274 *Pseudomonas* genomes.

Table S5. Carbohydrate Active EnZYmes (CAZYs) annotations of the representative proteins of the *Pseudomonas* pangenome.

Table S6. Annotations of the stress-resistance-related proteins of the representative sequences of the *Pseudomonas* pangenome.

Supplemental File 2. Datasheet including the *Pseudomonas* protein relationships with the isolation source based on Scoary analyses.

Supplemental File 3. Datasheet including the *Pseudomonas* Clusters of Orthologous Groups (COGs) relationships with the isolation source based on Scoary analyses.

Supplemental File 4. Datasheet including the *Pseudomonas* Carbohydrate Active enZYmes (CAZys) relationships with the isolation source based on Scoary analyses.

Supplemental File 5. Datasheet including the *Pseudomonas* resistance-related gene relationships with the isolation source based on Scoary analyses.

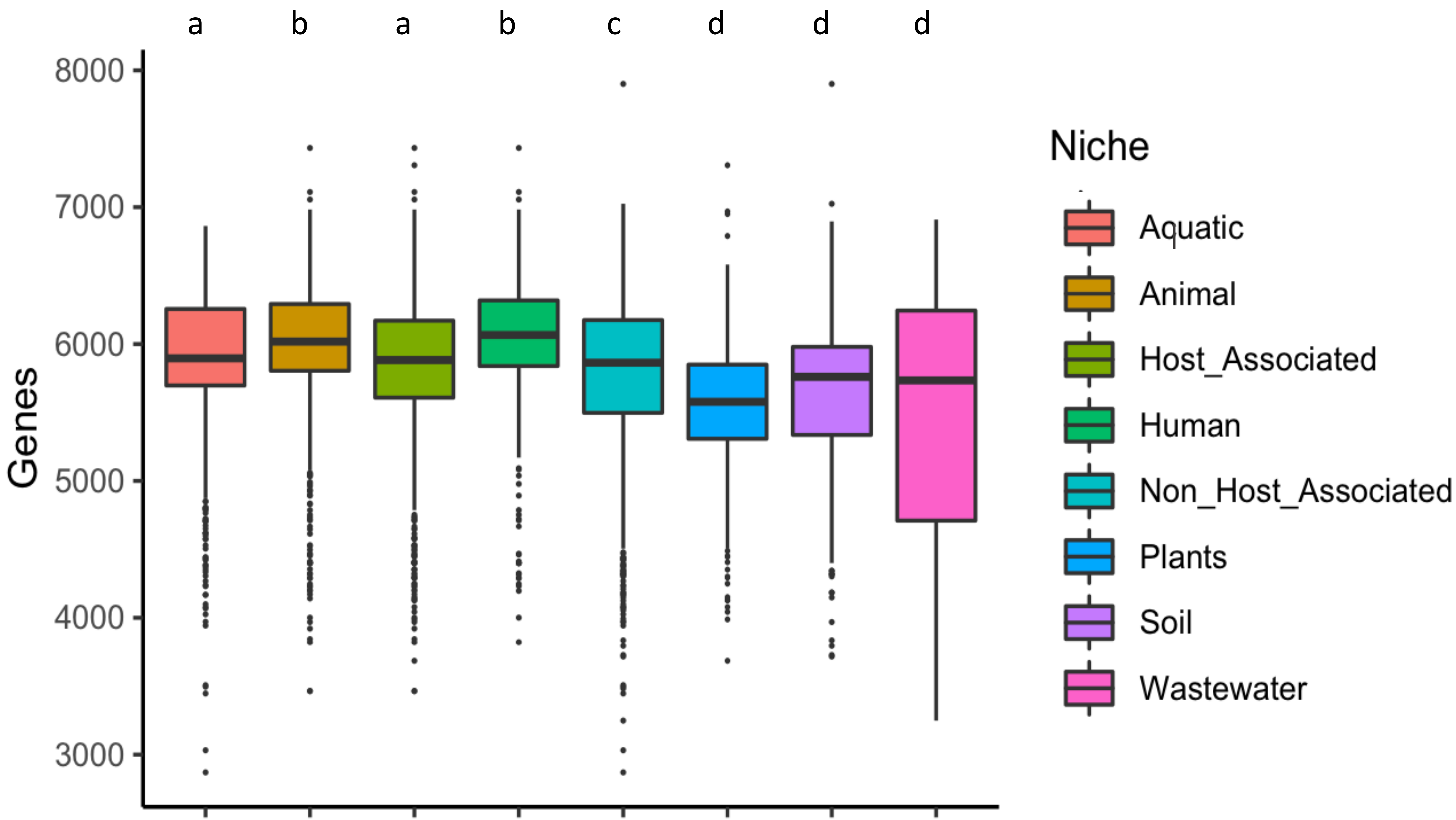
a
b
a
b
c
d
d
d
8000
7000
6000
5000
4000
3000
Genes
Niche
Aquatic
Animal
Host_Associated
Human
Non_Host_Associated
Plants
Soil
Wastewater

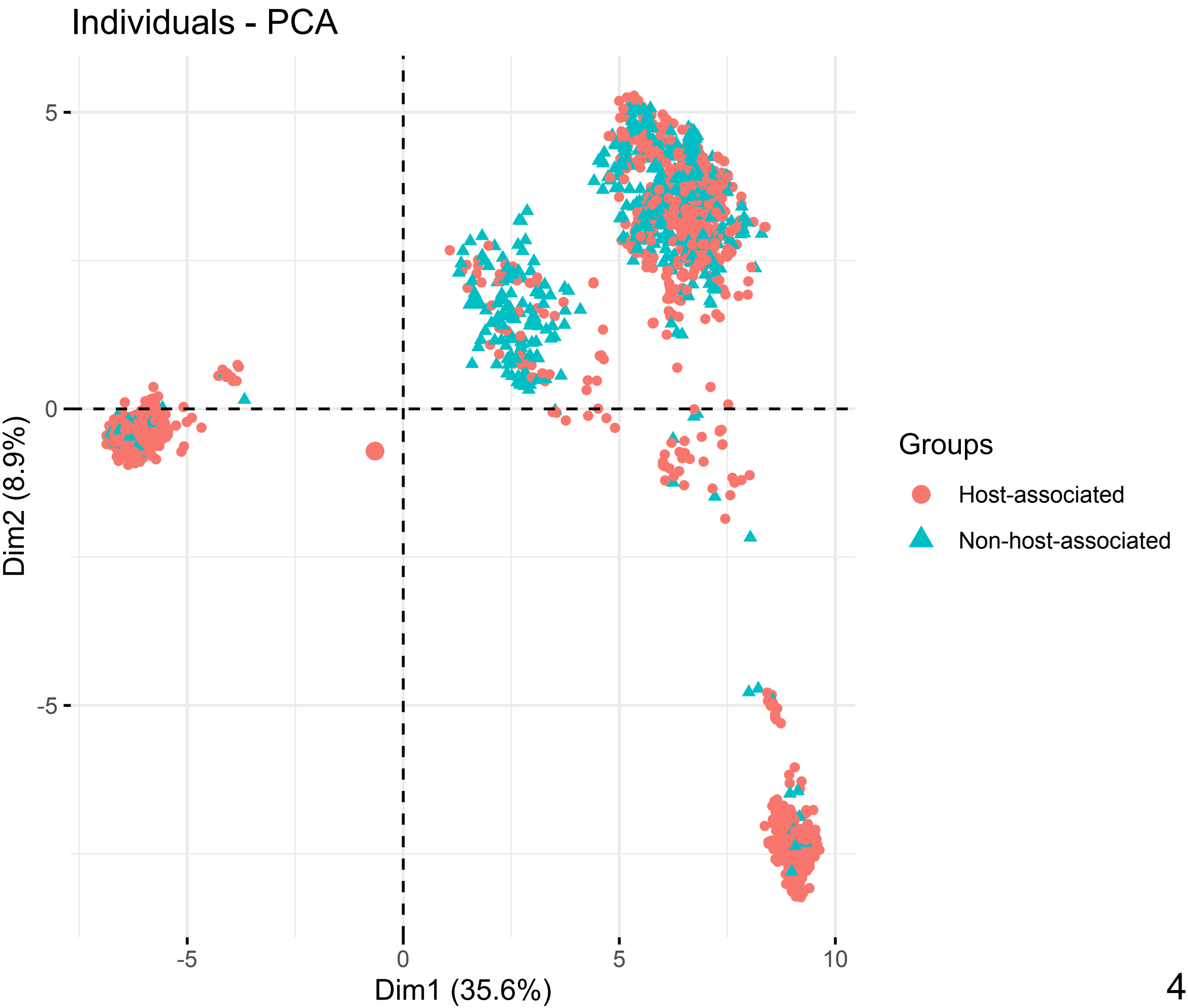

Individuals - PCA
Dim1 (35.6%)
Dim2 (8.9%)
-5
0
5
10
5
0
-5
Groups
Host-associated
Non-host-associated

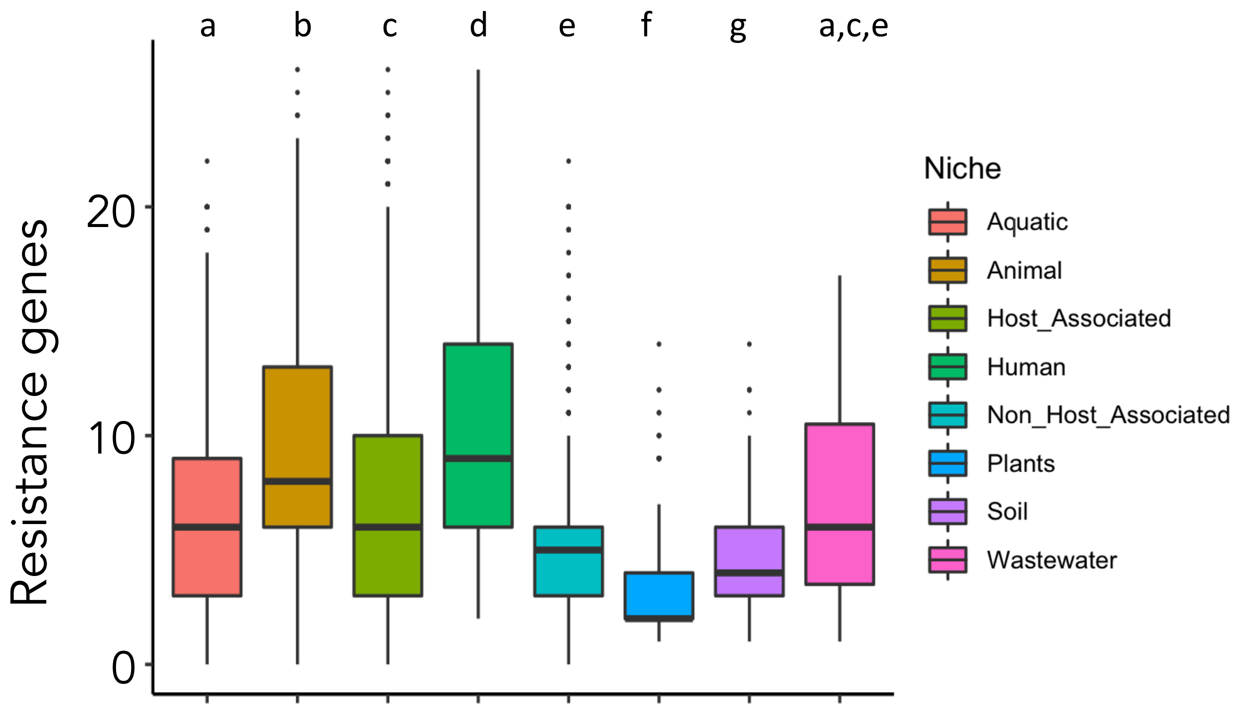

Resistance mechanisms			
Antibiotics	Aminoglycoside		51
	Beta-lactam		105
	Bleomycin		
	Efflux pumps		
		Fluoroquinolones	1
		Fosfomycin	6
		Glycopeptides	1
		Macrolides	5
	Phenicols		13
	Quinolones		2
	Rifamycins		4
	Streptogramins		1
	Sulfonamides		3
	Tetracyclines		4
	Sulfonamide		3
	Tetracycline		4
	Trimethoprim		1
Metals			34
Heat			7
Biocides			2

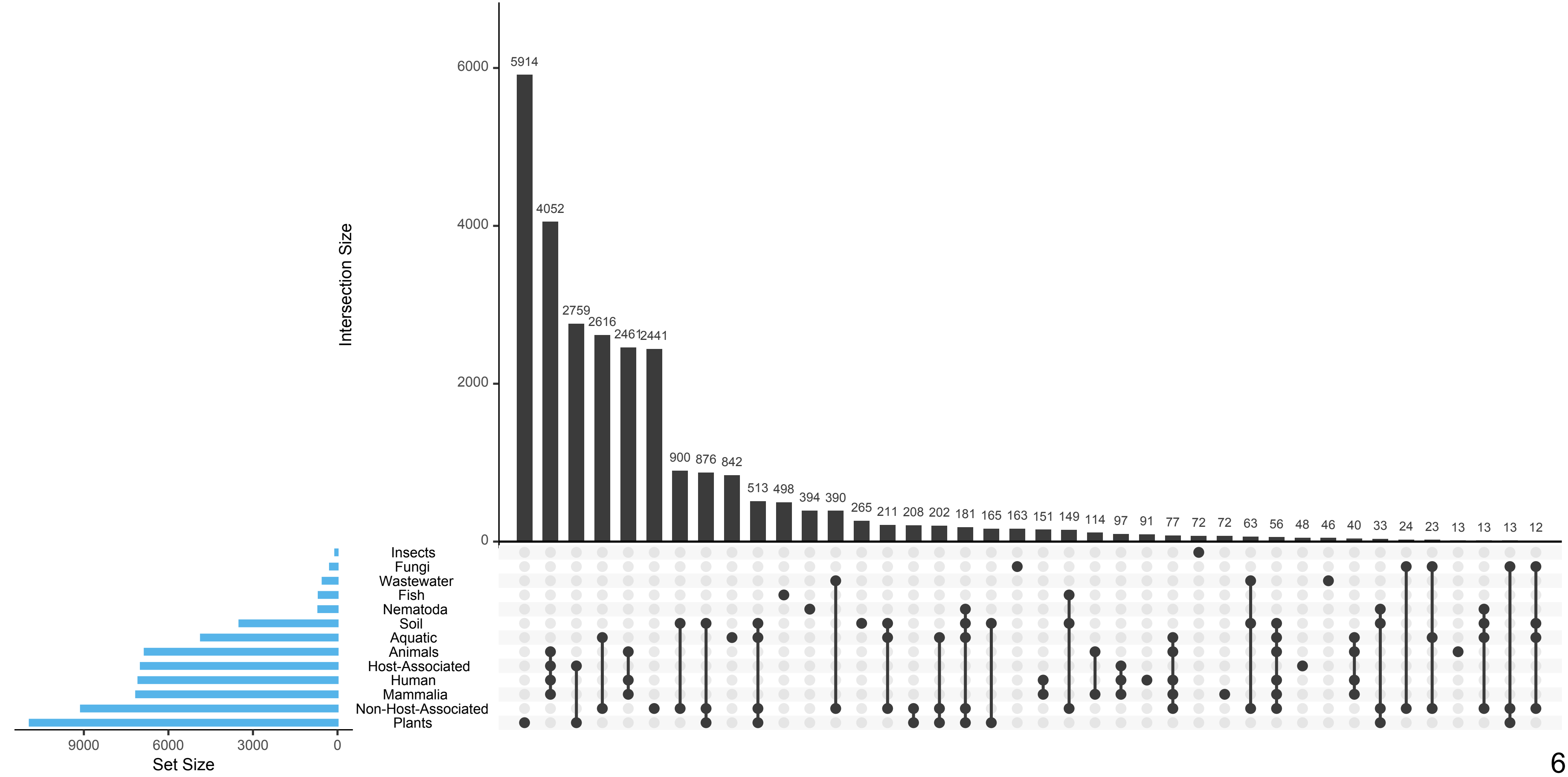
Intersection Size
6000
4000
2000
0
5914
4052
2759
2616
2461
2441
900
876
842
513
498
394
390
265
211
208
202
181
165
163
151
149
114
97
91
77
72
72
63
56
48
46
40
33
24
23
13
13
13
12
Insects
Fungi
Wastewater
Fish
Nematoda
Soil
Aquatic
Animals
Host-Associated
Human
Mammalia
Non-Host-Associated
Plants
9000
6000
3000
0
Set Size

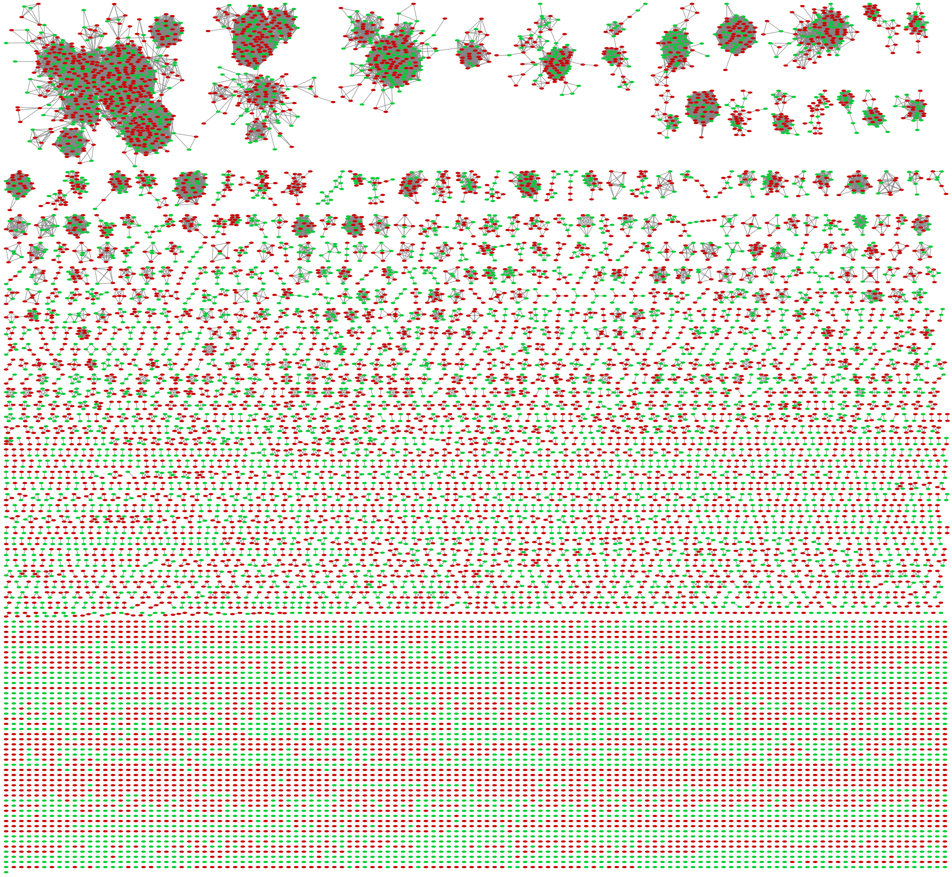

Supplement: Supplemental File 2 — Supplemental material. Download spectrum.02370-22-s0002.pdf, PDF file, 2.5 MB [file spectrum.02370-22-s0002.pdf]
